# Collapsing glomerulopathy in a young woman with *APOL1* risk alleles following acute parvovirus B19 infection: a case report investigation

**DOI:** 10.1186/s12882-016-0330-7

**Published:** 2016-09-06

**Authors:** Whitney Besse, Sherry Mansour, Karan Jatwani, Cynthia C. Nast, Ursula C. Brewster

**Affiliations:** 1 Government Medical College & Hospital Chandigarh Sector, Chandigarh, India; 2 Cedars-Sinai Medical Center, Los Angeles, California USA; 3 Section of Nephrology, Yale University, 330 Cedar Street, BB 121, New Haven, CT 06520-8029 USA

**Keywords:** Collapsing Glomerulopathy, FSGS, Parvovirus, Interferon, *APOL1*, Nephropathy, Immunohistochemistry

## Abstract

**Background:**

Collapsing Glomerulopathy (CG), also known as the collapsing variant of Focal Segmental Glomerulosclerosis (FSGS), is distinct in both its clinical severity and its pathophysiologic characteristics from other forms of FSGS. This lesion occurs disproportionally in patients carrying two *APOL1* risk alleles, and is the classic histologic lesion resulting from Human Immunodeficiency Virus (HIV) infection of podocytes. Other viral infections, including parvovirus B19, and drugs such as interferon that perturb the immune system, have also been associated with CG. Despite significant advances, explaining such genetic and immune/infectious associations with causative mechanisms and supporting evidence has proven challenging.

**Case presentation:**

We report the case of a healthy (HIV-negative) pregnant 36 year-old Caribbean-American woman who presented with nephrotic syndrome and fetal demise in the setting of acute parvovirus B19 infection. A series of three renal biopsies and rapid clinical course showed progression from significant podocyte injury with mild light microscopy findings to classic viral-associated CG to ESRD in less than 3 months. Genetic analysis revealed two *APOL1* G1 risk alleles.

**Conclusions:**

This is the first published case report of CG in the setting of acute parvovirus infection in a patient with two *APOL1* risk allelles, and parvoviral proteins identified in renal epithelium on kidney biopsy. These findings support the causative role of parvovirus B19 infection in the development of CG on the background of *APOL1* genetic risk.

**Electronic supplementary material:**

The online version of this article (doi:10.1186/s12882-016-0330-7) contains supplementary material, which is available to authorized users.

## Background

Collapsing Glomerulopathy (CG) represents approximately 12 % of focal segmental glomerulosclerosis (FSGS) histology and is more likely to present with nephrotic syndrome and rapid progression to end-stage renal disease (ESRD) compared with other FSGS variants [1, 2]. While other forms of FSGS have podocyte loss, glomerular epithelial cells in CG show evidence of hyperplasia and hypertrophy. In CG, there is a transition of typical markers of mature podocytes to an expression profile more characteristic of the parietal epithelial cells of Bowman’s capsule, raising questions of the origin of the abnormal glomerular epithelium [3]. These epithelial cells often appear swollen and vacuolated, with totally effaced foot processes overlying collapsed glomerular capillary loops, and may demonstrate mitotic activity. Extra-glomerular manifestations of CG, such as cystic tubular dilation, acute tubular injury and occasionally interstitial nephritis, are also commonly seen. Despite extensive research and some important advances in the field, the mechanisms resulting in this histologic lesion remain poorly understood, and thus targeted treatments do not yet exist.

Much of what is known about the pathogenesis of CG comes from human case reports and animal models. However, finding a central theme is difficult. Animal models suggest that HIV viral proteins, even when expressed only in podocytes, lead to both the glomerular and tubular characteristics of this lesion on particular genetic backgrounds [4]. Other animal models demonstrate CG resulting from seemingly unrelated mechanisms such as mitochondrial perturbations or oxidative stress [5]. Prior to the initiation of widely available and effective antiretroviral therapy, CG was the most common kidney lesion in HIV positive patients [6]. Importantly, other viruses besides HIV, particularly parvovirus B19, have been associated with CG [7, 8], as have medications such as interferon and pamidronate [5, 9]. Taken together, this multiplicity of causes leading to a unified phenotype highlights the need for further investigation of the common, necessary, and sufficient renal insults that result in this lesion.

The discovery of the association of *APOL1* genetic variants with FSGS and particularly HIV-associated Nephropathy (HIVAN) (odds ratio 17 and 29 respectively) has led to extensive effort to characterize the potential role of the protein product of *APOL1* [10]. This gene is a member of the APOL gene family that arose recently in evolution by gene duplication. It has been retained in humans, and some African primates, because of its role in lysis of trypanosomes [11]. The isoforms of APOL1 formed by the variants known as G1 and G2 seem to have the added ability to lyse *Trypanosoma rhodesiense* in addition to *Trypanosoma brucei* [11]. Its domain structure, which includes a transmembrane chloride channel, has been compared to bacterial colicins, diphtheria toxin, and bcl-2, each of which lead to cell death [12]. APOL1 is both a tissue and a secreted protein. Based on observations that *APOL1*-related risk is affected by the genotype of the donor kidney but not the recipient of a renal transplant [13], it seems that the tissue form is the one of relevance likely through cell toxic effects. Protein and mRNA localization reveal APOL1 expression in podocytes, glomerular endothelial cells, and tubular cells, but not mesangial cells [14]. There is no formal assessment in the literature of expression of APOL1 in parietal epithelial cells; however, images of protein and mRNA localization seem to show absence from the area of Bowman’s capsule [14, 15]. Notably, the penetrance of the “at-risk” *APOL1* genotype (homozygous or compound heterozygous for G1 or G2 allele) for kidney disease is much lower than that for other Mendelian diseases. Of patients with this at-risk genotype, only 4 % develop FSGS (however in the setting of untreated HIV infection, 50 % develop HIVAN) [10]. This low penetrance of the phenotype among patients with this genotype, coupled with undeniable association of the at-risk *APOL1* genotype with renal disease, suggests the need for a “second hit” as well as a likely role for other genetic or environmental factors. In this context, there may be a role of the “nephrotropic” parvovirus B19 in disease development.

Human parvovirus B19 is a small icosahedral ssDNA virus whose genome encodes two capsid proteins, VP1 and VP2, and a non-structural protein, NS1 [16]. Most adults worldwide show evidence of past infection, but symptoms vary from nearly asymptomatic to severe, depending on comorbidities and age at the time of primary infection [17]. Typically, immunocompetent adult hosts have very mild or asymptomatic disease, and are immune after antibody response to primary infection. Early symptoms of parvovirus infection result from death of erythroid lineage cells leading to reticulopenia. In susceptible hosts, this results in a transient aplastic anemia or hydrops fetalis. Later symptoms correspond to the development of an immune response to the virus, which may manifest as Fifth’s disease rash, gastrointestinal symptoms, or arthropathy [16]. Binding affinity of the capsid proteins for globoside receptors on erythroid-lineage cells leads to significant tropism of the virus for this cell type. Studies have since identified globoside Gb4, and similar glycosphingolipid receptors in numerous other tissue types including the kidney [18]_._ This finding adds plausibility to prior associations of hepatitis, myocarditis, and glomerulopathy with parvovirus infection made solely on temporal relation of viral infection and organ dysfunction coupled with the detection of parvoviral DNA in affected organ tissue.

Parvovirus B19 has been associated with CG for over 15 years, with several case reports demonstrating temporal relationship between infection and CG in both native and transplanted kidneys, but without definitive evidence of causality or explanation of its pathogenic role [8, 19, 20]. Parvoviral DNA was detected in archived kidney biopsies with a diagnosis of non-HIV related CG significantly more often than in biopsies of other kidney lesions [7, 8]. One of the two studies was able to localize this parvoviral DNA to visceral and parietal epithelial cells, as well as tubular cells. Seroprevalence (evidence of past exposure) was higher in patients with such lesions as well [8]. However, the detection of parvoviral DNA in approximately 25 % of both control samples and other glomerular lesions questions the specificity of this finding for disease pathogenesis. Experts in the field have suggested that showing evidence of parvoviral proteins in glomerular cells would help to prove infection by the virus in these cells as opposed to passive existence of the viral genome [21].

This case report hopes to direct attention to the role of the parvovirus B19 and *APOL1* interaction in CG.

## Case presentation

### Initial presentation

A previously healthy 36 year-old Caribbean woman, G4P2012, presented at 8 weeks gestation, with a 1-week history of worsening swelling in her hands and feet. Her prior pregnancies were without complication, and she took no medications other than prenatal vitamins. She had no personal or family history of kidney disease or immunodeficiency. Her prenatal workup was unremarkable and HIV test negative. Her physical exam on presentation was significant for a blood pressure of 146/95 mmHg, and 3+ pitting edema up to her knees. Approximately 1 month prior to presentation, her 4-year-old daughter had experienced a classic presentation of “Fifth disease” from parvovirus B19. On admission, an obstetrical ultrasound revealed fetal demise and she underwent a dilation and curettage procedure. Products of conception were not sent for parvovirus staining as the obstetrical team felt her presentation was entirely consistent with acute parvovirus infection.

Notable laboratory and imaging results included serum creatinine concentration (SCr) of 557 umol/L (6.3 mg/dL), blood urea nitrogen of 15.3 mmol/l (43 mg/dL), serum albumin 15 g/L (1.5 g/dL), total cholesterol 10.55 mmol/L (408 mg/dL), triglycerides 7.01 mmol/L (621 mg/dL), LDL 7.86 mmol/L (304 mg/dL), INR 0.96, PTT 33 s, and PT 10.7 s. A spot urine protein:creatinine ratio was 20.8 g/g. Urine microscopy revealed 2–5 white blood cells per high power field and maltese crosses were diffusely present upon polarization. Further serologic workup was negative, including ANA, anti-dsDNA, repeat HIV test, and for hepatitis B and C. Serum parvovirus B19 qPCR detected the parvoviral genome with a modest viral load of approximately 2000 copies, and parvovirus serologies both IgM and IgG were elevated demonstrating acute infection. While, “undetected” would be the reported result for unexposed individuals, the clinical virology lab reports that their sensitive qPCR assay allows for viral load quantification from approximately 200 copies to over 1X10^10^ (with the higher values only seen in immunocompromised and sickle cell patients who present early and without adequate immune response). Renal ultrasound revealed normal sized kidneys bilaterally and slight increase in echogenicity, but without hydronephrosis, cysts, or calcifications.

### Renal biopsy/diagnosis

**Fig. 1:**
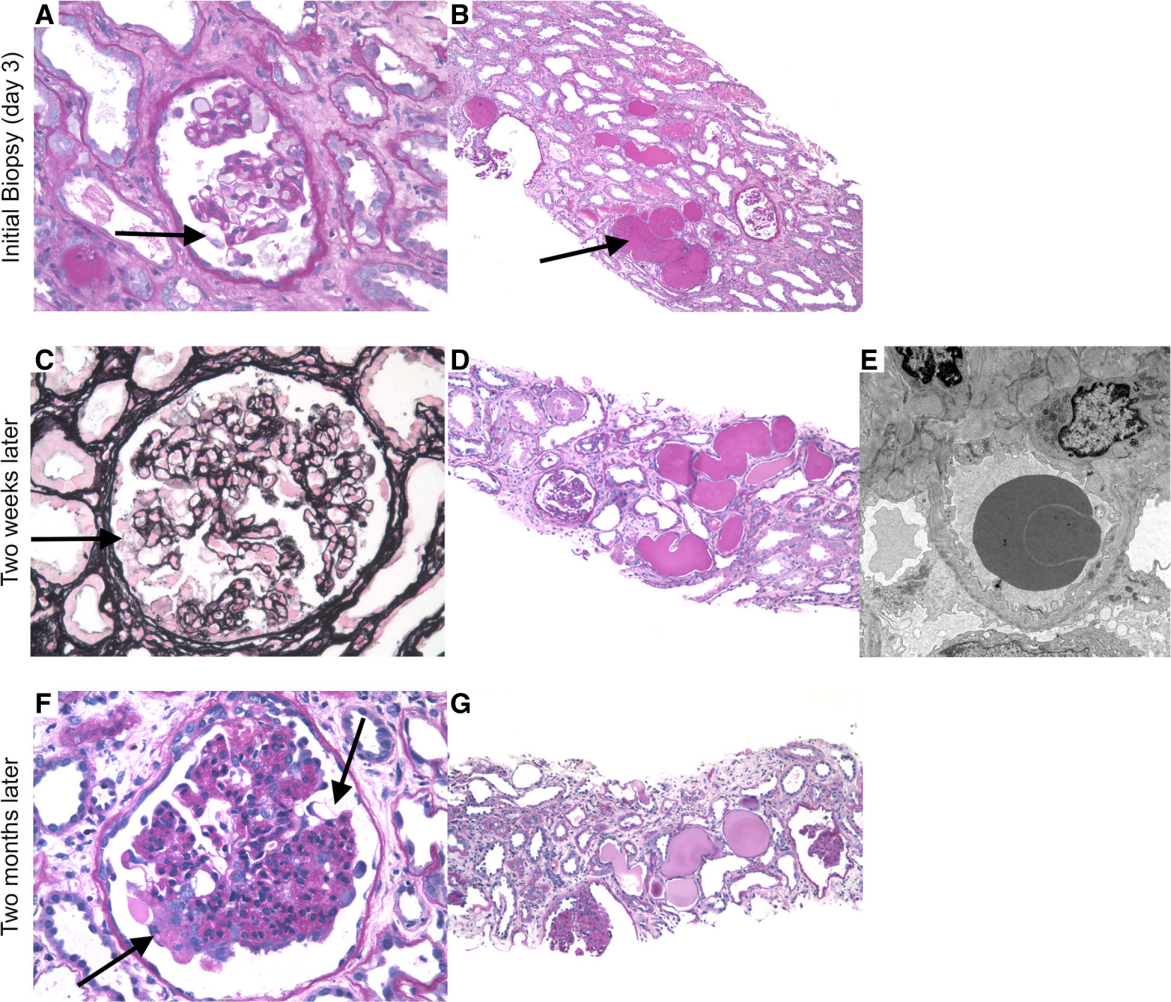
Kidney biopsy images from initial presentation (**a** – 40x, **b** – 10x PAS), 2 weeks later (**c** – 40x Jones, **d** – 10x PAS, **e** – EM 8000x) and 2 months later (**f** – 40x, **g** – 10x PAS). At initial presentation, glomeruli have patent capillary loops with few hypertrophic vacuolated podocytes (**a** - *arrow*). There is acute tubular injury and clusters of microcystically dilated tubules containing proteinaceous casts (**b** - *arrow*). Two weeks later, glomeruli show more pronounced collapsing features with prominent vacuolated podocytes and capillary obliteration (**c** - *arrow*). Microcystically dilated tubules remain with acute tubular injury and an increase in tubulointerstitial scarring (**d**). Electron microscopy demonstrates extensive foot process effacement (**e**) without electron dense deposits or tubuloreticular inclusions. Two months after initial presentation, advanced glomerular collapse and sclerosis are present with persistent focal and segmental epithelial cell hypertrophy and hyperplasia (**f** - *arrow*), and dilated tubules with progressive tubulointerstitial scarring (**g**)

**Fig. 2:**
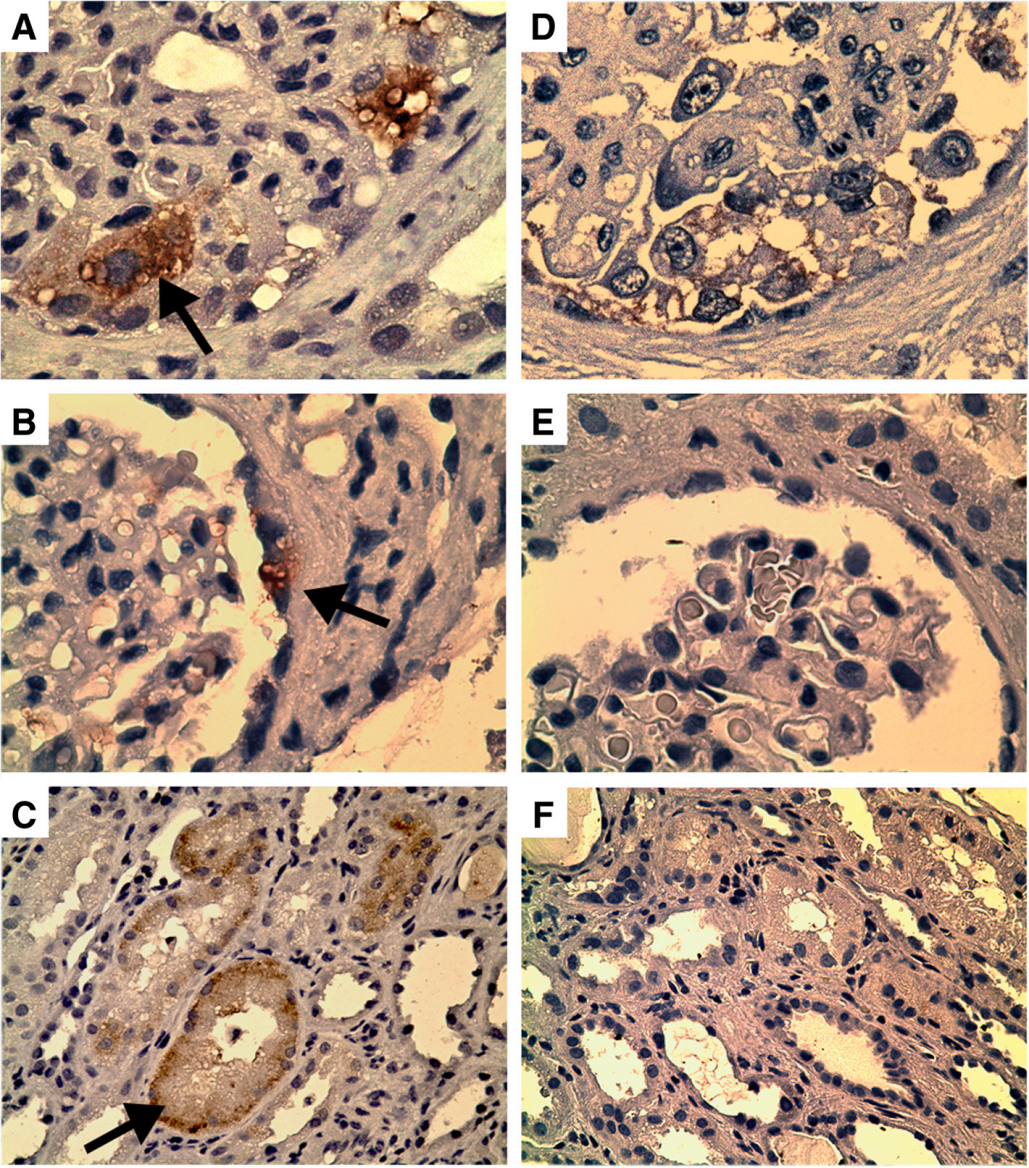
*Parvovirus Capsid Protein Staining*. Immunohistochemistry results on patient’s initial renal biopsy (day 3 of presentation) using primary antibody to human parvovirus B19 capsid proteins VP1 and VP2 and anti-mouse-HRP secondary antibody. Strong positive staining indicated by arrows is noted in the cytoplasm of hypertrophic podocytes (**a** original magnification x100), parietal epithelial cells (**b** original magnification x 100), and tubular epithelium (**c** original magnification x 40). Negative staining in hyperplastic podocytes from a different case of collapsing glomerulopathy associated with anabolic steroid use, stained as a negative control (**d** original magnification x 100). HIVAN and lupus associated collapsing podocytopathy cases also stained negatively for parvovirus B19 (data not shown). Non-specific mouse IgG with same anti-mouse-HRP was used on additional section from patient’s same biopsy as a negative control and was negative throughout the biopsy specimen (**e** original magnification x100, **f** original magnification 40x)

The patient underwent renal biopsy (Fig. 1) on day 3 of presentation, with a limited biopsy sample revealing 10 mostly unremarkable glomeruli aside from few swollen and vacuolated podocytes found focally and segmentally. There was acute tubular injury with simplification of tubular epithelium, loss of brush border staining, and dilated tubular lumina, as well as a moderate interstitial inflammatory infiltrate without significant tubulointerstitial scarring. Immunofluorescence was not done due to sample inadequacy. Electron microscopy demonstrated patent capillary loops with minor basement membrane irregularity, but no immune complex deposits or tubuloreticular inclusions. There was global podocyte foot process effacement. Immunohistochemistry for parvoviral proteins (Cell Marque R29F6 mouse monoclonal antibody [22, 23], 1:100 dilution, with appropriate negative controls) was performed after the time of clinical care, and demonstrated positivity in hypertrophic podocytes, parietal epithelial cells, and tubular cells (Fig. 2). Given her clinical diagnosis of acute parvovirus infection, and the glomerular changes along with the microcystically dilated tubules, the biopsy was interpreted as showing changes suggestive of Collapsing Glomerulopathy with acute tubular injury and mild active interstitial nephritis. The hope was that, if attributable to parvovirus, this injury would improve with self-limited resolution of viral infection over time. She was treated with oral prednisone (60 mg daily) for the interstitial nephritis. Her SCr did not improve, and peripheral edema worsened despite diuretics. Two weeks later, she was re-biopsied to determine a more definitive diagnosis. A total of nine glomeruli were present, two segmentally sclerotic with more pronounced podocyte hypertrophy and hyperplasia, and prominent microcystically dilated tubules. Immunofluorescence microscopy was performed and was negative for immune reactants. A diagnosis of CG, likely virally-mediated, was made.

### Clinical progression

She was discharged home after 3 weeks of hospitalization, with tapering prednisone and an aggressive diuretic regimen. She returned 6 days later with orthostatic hypotension despite persistent anasarca (80+ pounds above her baseline). Her serum albumin was 10 g/L (1.0 g/dL). Water immersion therapy with diuresis was attempted in an effort to alleviate the patient’s edema, but had little effect.

Her SCr had improved to 309 umol/L (3.5 mg/dl) by 5 weeks after presentation, but she then developed pancytopenia. Serum hemoglobin fell to 50 g/L (5 g/dL), platelet count to 15,000/uL, and ANC as low as 1700/uL with an inadequate rise in reticulocyte count. Serum lactate dehydrogenase and haptoglobin remained normal. Bone marrow biopsy showed a markedly hypocellular marrow with mild dyserythropoesis. While no viral inclusions or hemophagocystosis were found, her serum ferritin rose to 2260 ug/L. This was proposed to be from parvovirus-related inflammation, which has been reported previously [24]. Repeat parvoviral testing showed persistently detectable low levels of virus in the 200–1000 copy range. Intravenous immunoglobulin (IVIG) was started for multi-organ complications of parvovirus, despite low copy numbers. Preparations of IVIG contain parvovirus specific antibodies at concentrations capable of neutralizing parvoviral antigens [23, 25] and have been shown to be helpful in eradicating parvovirus B19-associated anemia [26, 27]. IVIG dosing was empiric, and notably, most of the infusion was lost in the urine (proteinuria up to 60 g/day following infusion). Dosing was continued at 1 g/kg every other day, and eventually quantitative PCR showed estimated viral copy number less than 200 copies, although persistently detected. Because she had no initial response to the IVIG and her pancytopenia was severe, she also received intravenous solumedrol (60 mg daily for 2 weeks, then tapering). After a total of 1 month of hematologic issues, cell counts returned to normal. Initially after beginning IVIG treatment, the SCr reached a nadir of 256 umol/L (2.9 mg/dl), but with her worsening pancytopenia, kidney function worsened, and SCr rose to over 530 umol/L (6 mg/dl).

A third renal biopsy was undertaken 10.5 weeks after initial presentation to help decide whether to consider cytotoxic therapy for FSGS in a now fairly debilitated young woman. Due to the extent of disease progression on this biopsy, the decision was made to forego any cytotoxic therapy and to initiate dialysis for uremia and volume management.

### Genetic analysis

Given the uncommon severity of this case and the possibility of African ancestry given her Caribbean origin [28], the possibility of a genetic susceptibility to podocyte injury was considered. To investigate this, venous blood was collected in the setting of an IRB-approved research protocol and genomic DNA was extracted and sent for whole exome sequencing by Illumina HiSeq 2000 technology. The authors used well validated scripts made available by the Yale Genetics/Yale Center for Genomic Analysis [29] to curate, align, and annotate the sequencing data. Mean coverage was 57.4 reads, and there was 92.4 % 10x coverage. As hypothesized may be the case, our patient carries two *APOL1* risk alleles (homozygous missense variants p.S342G and p.I384M which together comprise the G1 risk allele). For completeness, the following 50 additional genes with published association with glomerular diseases were also assessed: *ACTN4*, *ADCK4*, *ALG1*, *ALMS1*, *ANLN*, *APOA1*, *APOL1*, *ARHGAP24*, *ARHGDIA*, *ATGB4*, *B2M*, *CD151*, *CD2AP*, *CFH*, *COL4A3*, *COL4A4*, *COQ2*, *COQ6*, *CRB2*, *CUBN*, *DGKE1*, *EMP2*, *FGA*, *FN1*, *GLA*, *INF2*, *ITGA3*, *ITGB4*, *LAMB2*, *LMX1B*, *LYZ*, *MEFV*, *MYH9*, *MYO1E*, *NEIL1*, *NLRP3*, *NPHS1*, *NPHS2*, *PAX2*, *PDSS2*, *PLCE1*, *PLCG2*, *PMM2*, *PTPRO*, *SCARB2*, *SMARCAL1*, *TRPC6*, *WDR73*, *WT1*, *ZMPSTE24*. There were no loss of function (nonsense, frameshift, splice site) variants, nor were there any rare non-synonomous variants with predicted deleterious consequence in these genes.

### Subsequent follow-up

Over the subsequent 2 months on hemodialysis, the patient became anuric. Her serum albumin climbed to 3.0 g/L (by 5 months after her initial presentation). She was evaluated for transplantation, and successfully received a living donor kidney transplant from her sister 8.5 months after initial presentation and 6 months after starting hemodialysis. Our findings of the patient’s *APOL1* genotype were made available to transplant physicians during transplant evaluation and clinical genetic testing was suggested to the patient’s donor (sister) but she declined. As there are no established guidelines in place for *APOL1* testing of donor organs, the donation was accepted. With over 6 months of follow-up, her renal function fortunately remains excellent, with <500 mg proteinuria, and no anemia.

## Discussion/Conclusions

This case report adhered to CARE (Additional file 1) guidelines for reporting case reports. This case broadens existing evidence that acute parvovirus infection can lead to CG in individuals at risk due to *APOL1* genotype. It remains to be seen if the *APOL1* at-risk genotype is essential for development of this lesion following parvovirus infection. Importantly, CG is not the only renal lesion seen in those with the at-risk *APOL1* risk genotype [30], so other genetic or environmental factors likely play a role in determining what renal lesion, if any, a particular patient will get. While it was initially difficult to describe a unifying explanation for all of the human and animal model findings regarding the pathogenesis of CG, the potential interaction between viruses and APOL1 may finally be coming into focus. There are accumulating data suggesting that the interferon-mediated response to viruses may increase production of a cell-toxic APOL1 variant protein.

Eleven cases of CG following interferon treatment given for a different medical conditions have been reported [9], and genotyping recently was provided for 7 of these patients, all of whom carried two *APOL1* risk alleles [31]. Nichols et al characterized this association further by demonstrating that interferon exposure up-regulates wild-type and risk-variant *APOL1* transcription, including production of alternative transcripts [31]. Moreover, HEK cells transfected with G1 or G2 genotypes of *APOL1* transcripts showed increased cytotoxicity/viability ratio compared to the wild-type genotype. Indeed, other investigators have found that risk variant APOL1 proteins have cell lytic capabilities due to lysosomal membrane pore forming function perturbing vesicular trafficking, and could cause podocyte injury in vitro and hepatocyte necrosis in vivo [32]. Taken together, these findings suggest that interferon stimulation in a patient with *APOL1* risk alleles leads to increased production of a cell-toxic protein [31]. The proposed connection of this finding with viral infection is the interferon. Similar to the immune response for many viruses [33], review of the literature suggests that the typical immune response to parvovirus involves a necessary and lasting antibody response to parvoviral capsid proteins as well as recruitment of a T_H_1 T-cell response, which includes production of cytokines such as interferon gamma [34–36]. While this hypothesis is appealing, it seems that with >10 % of African Americans carrying this at-risk *APOL1* genotype, simple systemic immune response to relatively common viral infections should make CG more prevalent than it is [37]. It is also important to note that Divers et al [38] showed that exposure to JC virus had a protective influence in patients with *APOL1* risk variants [39], so not all viruses have the same effect. Likely an exaggerated immune response, additional kidney insults, or more direct kidney targeting (i.e. direct infection of podocytes by parvovirus or HIV) of immune responses is necessary. The identification of parvoviral proteins in affected renal epithelial cells (Fig. 2) demonstrates viral transcription/translation rather than just passive existence of genomic material. This supports a role for direct viral infection of these cells preceding renal disease.

Our patient’s immune response to parvovirus may have been atypical. Firstly, her significant associated symptoms (hydrops fetalis, pancytopenia, and CG) are more severe than expected in an immunocompetent host. Secondly, this occurred in the setting of a low viral load during acute infection. Her viral load failed to clear promptly; however, it has been suggested that parvovirus DNA can be detected for months to years after acute infection in a normal person [16]. Worth noting is the potential role pregnancy may play on immune modulation in this case. Furthermore, while our hematology consultants were left puzzled as to the precise etiology of her profound pancytopenia that lasted several weeks, they considered it to be an inflammatory reaction in the spectrum of Hemophagocytic Lymphohistiocytosis, a disease process in which interferon gamma production is thought to be central.

This case provides evidence of parvoviral proteins in renal epithelial cells and a strong case for the role of parvovirus B19 infection in the pathogenesis of CG in a patient genotyped to show homozygous *APOL1* G1 risk alleles. We believe that with parvovirus B19 infection identified as one potential “second hit” in those with *APOL1* risk alleles, perhaps clinical consideration of silent infection in other cases of “idiopathic” CG should be considered to aid in further data accumulation on this fascinating topic.

## Abbreviations

ANA, antinuclear antibody; dsDNA, double-stranded DNA; ESRD, End Stage Renal Disease; FSGS, Focal Segmental Glomerulosclerosis; HIV, Human Immunodeficiency Virus; HIVAN, HIV-associated Nephropathy; qPCR, quantitative polymerase chain reaction; SCr, serum creatinine

## Additional file

Additional file 1: CARE Checklist—2016: Information for writing a case report. (DOCX 601 kb)
