# Supplementary material for: Collapsing glomerulopathy in a young woman with APOL1 risk alleles following acute parvovirus B19 infection: a case report investigation
Source: BMC Nephrol. 2016 Sep 6;17(1):125. doi: 10.1186/s12882-016-0330-7 (PMC5013576; doi:10.1186/s12882-016-0330-7)
Supplement: Additional file 1: — CARE Checklist—2016: Information for writing a case report. (DOCX 601 kb) [file 12882_2016_330_MOESM1_ESM.docx]

**CARE Checklist – 2016: Information for writing a case report**

**Topic Item Checklist item description Line/Page**

**Title 1** The words “case report” should be in the title along with the area of focus __3/1____

**Key Words 2** Four to seven key words—include “case report” as one of the key words __25/1____

**Abstract 3a** Background: What does this case report add to the medical literature? ___3/2___

**3b** Case summary: chief complaint, diagnoses, interventions, and outcomes ___13/2___

**3c** Conclusion: What is the main “take-away” lesson from this case? ___20/2___

**Introduction 4** The current standard of care and contributions of this case—with references (1-2 paragraphs) __2/3, 6__

**Timeline 5** Information from this case report organized into a timeline (table or figure) __fig 1 1\\___

**Patient Information 6a** De-identified demographic and other patient or client specific information ___23/6___

**6b** Chief complaint—what prompted this visit? ___24/6___

**6c** Relevant history including past interventions and outcomes ___2/7__

**Physical Exam 7** Relevant physical examination findings ___4/7___

**Diagnostic 8a** Evaluations such as surveys, laboratory testing, imaging, etc. ___7/9__

**Assessment 8b** Diagnostic reasoning including other diagnoses considered and challenges ___7/11_

**8c** Consider tables or figures linking assessment, diagnoses and interventions _8:Fig.1&2_

**8d** Prognostic characteristics where applicable ___n/a__

**Interventions 9a** Types such as life-style recommendations, treatments, medications, surgery __8/11_

**9b** Intervention administration such as dosage, frequency and duration __8/11_

**9c** Note changes in intervention with explanation __22-8__

**9d** Other concurrent interventions 7/8, 19/9__

**Follow-up and 10a** Clinician assessment (and patient or client assessed outcomes when appropriate) __9/11__

**Outcomes 10b** Important follow-up diagnostic evaluations __9/11__

**10c** Assessment of intervention adherence and tolerability, including adverse events __11____

**Discussion 11a** Strengths and limitations in your approach to this case __12/14_

**11b** Specify how this case report informs practice or Clinical Practice Guidelines (CPG) __12/14_

**11c** How does this case report suggest a testable hypothesis? __14____

**11d** Conclusions and rationale __14____

**Patient Perspective 12** When appropriate include the assessment of the patient or client on this episode of care __n/a____

**Informed Consent 13** Informed consent from the person who is the subject of this case report is required by most journals __obtained.

**Additional Information 14** Acknowledgement section; Competing Interests; IRB approval when required ___18___
